# Comparative analysis of HiSeq3000 and BGISEQ-500 sequencing platform with shotgun metagenomic sequencing data

**DOI:** 10.5808/gi.23072

**Published:** 2023-12-29

**Authors:** Animesh Kumar, Espen M. Robertsen, Nils P. Willassen, Juan Fu, Erik Hjerde

**Affiliations:** 1 Center for Bioinformatics, Department of Chemistry, UiT The Arctic University of Norway, Tromsø, 9037, Norway; 2 Faculty of Biosciences, Department of Livestock and Aquaculture Science, Norwegian University of Life Sciences, Ås 1433, Norway

**Keywords:** benchmarking, BGISEQ-500, HiSeq3000, metagenome

## Abstract

Recent advances in sequencing technologies and platforms have enabled to generate metagenomics sequences using different sequencing platforms. In this study, we analyzed and compared shotgun metagenomic sequences generated by HiSeq3000 and BGISEQ-500 platforms from 12 sediment samples collected across the Norwegian coast. Metagenomics DNA sequences were normalized to an equal number of bases for both platforms and further evaluated by using different taxonomic classifiers, reference databases, and assemblers. Normalized BGISEQ-500 sequences retained more reads and base counts after preprocessing, while a slightly higher fraction of HiSeq3000 sequences were taxonomically classified. Kaiju classified a higher percentage of reads relative to Kraken2 for both platforms, and comparison of reference database for taxonomic classification showed that MAR database outperformed RefSeq. Assembly using MEGAHIT produced longer assemblies and higher total contigs count in majority of HiSeq3000 samples than using metaSPAdes, but the assembly statistics notably improved with unprocessed or normalized reads. Our results indicate that both platforms perform comparably in terms of the percentage of taxonomically classified reads and assembled contig statistics for metagenomics samples. This study provides valuable insights for researchers in selecting an appropriate sequencing platform and bioinformatics pipeline for their metagenomics studies.

## Introduction

Technological advancements and improvements in next-generation sequencing have allowed for the exploration of complex and previously unknown marine microbial communities, which constitute the largest and most stable ecosystem on earth. Studies such as the *Tara Oceans* project [1] have enabled the discovery of so-called microbial dark matter [2] or unculturable microbial communities. The main challenge in metagenomics study is to identify low-abundance microbes, which highly depends on the accuracy and precision of short-reads sequencing platforms. While short reads may introduce sequencing errors, high coverage can compensate these errors in subsequent downstream analysis [3]. Short read sequencing platform such as Illumina HiSeq3000 platform can generate 1 tera-basepairs (Tbp) of sequence data which corresponds to 3.33 billion paired-end (PE) reads (150 bp) in a single run and is based on sequencing by synthesis chemistry. It has already been the preferred choice for shotgun metagenomics studies due to their affordability and low error rates [4].

The BGISEQ-500 platform, based on DNA Nanoball Technology, by the Beijing Genomics Institute (BGI) group in 2016, generates 1 Tbp of sequence data which corresponds to 5 million PE reads (100 bp) in a single run while minimizing amplification errors. DNA Nanoball Technology incorporates customized combined probe-anchor synthesis technology with MGI Tech Co., Ltd's proprietary base-calling software. Several studies have compared the BGI’s platform performance with Illumina’s platform in different areas of omics studies such as transcriptome, small RNA sequencing including metagenomics studies [5-7].

One of the key challenges in metagenomics studies utilizing short-read platforms is that short-read sequences often map to multiple species with identical or similar segments in the reference genome [8]. To address this issue, taxonomic classifiers (bioinformatics tools), utilize specialized algorithms to accurately assign millions of reads generated from short read sequencers to the corresponding taxa [9]. Taxonomic classifiers such as Kraken2 [10] use both least common ancestor (LCA) and k-mer approaches to build an indexed database and searches for k-mers in the reads that match against the reference database. Whereas other taxonomic classifiers such as Kaiju [11] perform maximum exact match against protein databases using Burrows-Wheeler transform. Both k-mer based classifiers are designed for short reads and utilize pseudo-alignment algorithms to match them against a reference database for classification.

However, a downside of this method is the reliability of taxonomically annotated reference sequences database as uncharacterized taxa generally lead to insufficient classification at the *species* level [12]. Highly accurate relevant reference sequence databases, such as the NCBI RefSeq database [13] and the MAR databases (https://mmp2.sfb.uit.no/) [14] can accelerate taxonomic profiling of marine metagenomics reads or contigs. The MAR databases hosted at the Center for Bioinformatics (SfB), The Arctic University of Norway (UiT), a node of ELIXIR Norway, contain marine microbial genome records based on the level of completeness (MarRef v1.5: 1270 manually curated records and MarDb v1.5: 13237 incompletely sequenced marine prokaryotic genomes records including metagenome-assembled genomes [MAGs] and single amplified genomes [SAGs]). These records are taxonomically annotated using both Genome Taxonomy Database (GTDB) [15] and NCBI, allowing flexibility to use either GTDB taxonomy or NCBI taxonomy identifier (TaxID). Both GTDB and NCBI taxonomy are hierarchically ordered into taxonomic levels or ranks. The most commonly used taxonomic ranks for bacteria include domain/kingdom, phylum, class, order, family, genus, and species.

In this study, we provide an extensive comparison of sequencing platforms (HiSeq3000 and BGISEQ-500) using 12 sediment metagenomics samples, utilizing various combinations of taxonomy classifiers with reference databases and assemblers. By doing so, this study aims to provide insights into the advantages and limitations of each platform and contribute to the ongoing efforts to improve and optimize metagenomics research.

## Methods

### Metagenomic DNA extraction

The study collected marine sediment samples from 12 locations off the Norwegian coast and metagenomic DNA was extracted using the FastDNA Spin Kit for Soil (MP Biomedicals, California, USA). The DNA samples were sequenced using both HiSeq3000 (Norwegian Sequencing Centre, Oslo, Norway) and BGISEQ-500 (BGI Tech Solutions (Hong Kong) Co., Ltd., Hong Kong, China) platforms.

### Data available

All the sequencing reads generated from both HiSeq3000, and BGISEQ-500 platforms have been deposited in the European Nucleotide Archive (ENA) at EMBL-EBI under accession number PRJEB55540. A copy of the BGISEQ-500 generated sequences has also been submitted to the CNGB Sequence Archive (CNSA) of China National GeneBank DataBase (CNGBdb) with accession number CNP0003834.

### Normalization and preprocessing of sequence data

**Fig. 1.**
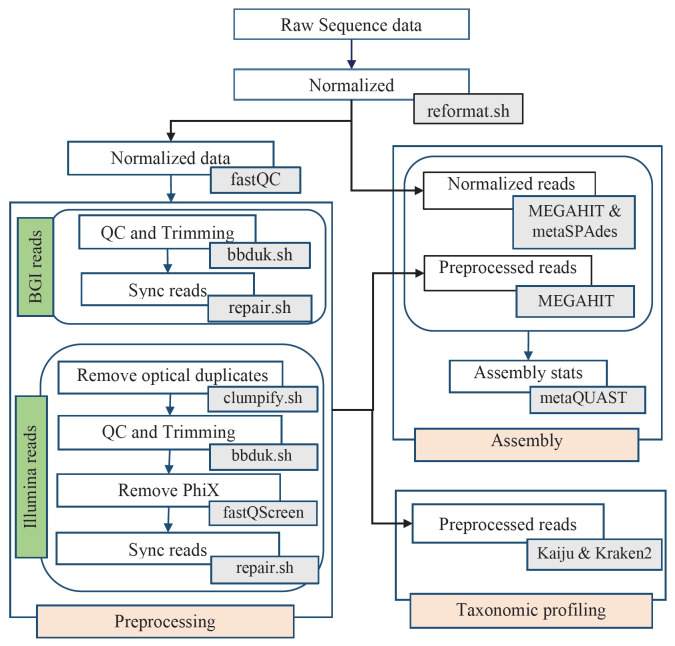
Workflow for analyzing HiSeq3000 and BGISEQ-500 generated data. Major analysis steps are indicated in light red (Preprocessing, Assembly, and Taxonomic classification). Arrows indicate the direction of data flow. Tools used in each analysis step are indicated in gray boxes.

**Fig. 2.**
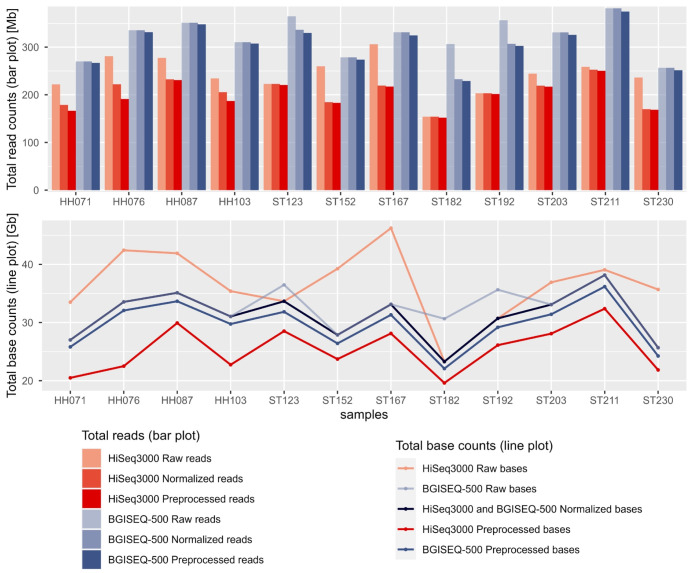
Total read count (bar plot) and total base counts (line plot) present in raw, normalized, and preprocessed read sequences generated from two sequencing platforms. Sequences in red and blue variants represent HiSeq3000 and BGISEQ-500 total reads and bases counts, respectively, while the black line graph represents normalized base counts for both sequencing platforms.

In this study, we divided each 12 samples into two, treating them as technical replicates (Illumina HiSeq3000: 150 bp PE and BGISEQ-500: 100 bp PE; Illumina, San Diego, CA, USA). To normalize sequence data, the largest dataset from each pairwise technical replicate was normalized (downsampled) to an equal number of bases per sample points for accurate comparison. It was done using reformat.sh of the BBTools suite (sourceforge.net/projects/bbmap/) with the "*sbt*" option (*sbt*: ‘lowest base count of sequences generated among sequencing platforms per sample site’) (Figs. 1 and 2). The normalized or unprocessed reads were then screened for overall sequencing quality using FastQC v0.11.5 [16] and optical duplicates for Illumina sequences were removed using clumpify.sh (dupedist = 40) of the BBTools package. Adapter sequences were trimmed using bbduk.sh (HiSeq3000 = Nextera adapter sequences, BGISEQ-500 adapter sequences left = ‘*AAGTCGGAGGCCAAGCGGTCTTAGGAAGACAA*’ and right = ‘*AAGTCGGATCGTAGCCATGTCGTTCTGTGAGCCAAGGAGTTG*’) and low-quality bases were filtered out (HiSeq3000: ‘forcetrimleft = 17 ktrim = r minlen = 51 qtrim = r trimq = 20 tbo = t mink = 11 hdist = 1’ and BGISEQ-500: ‘minlen = 51 trimq = 20 forcetrimleft = 3’). Trimmed reads were mapped against PhiX phage sequences using FastQ Screen [17] to filter any possible contamination in Illumina sequences. Finally, PE reads were reordered using repair.sh and clean reads greater than 51 bases with Phred score > 20 were kept for downstream analysis.

### Taxonomic classification

Taxonomic classification of the preprocessed or clean reads was performed using Kaiju (v1.7.3) and Kraken2 (v2.1.0) against both indexed bacterial MAR (v1.5, June 2020) and RefSeq (July 2020) databases at default parameter settings and *thread 15*. The MAR databases differentiate marine microbial genomes based on the level of curatedness. In the current study, both MarRef v1.5 and MarDb v1.5 were merged (here referred to as MAR) and used as the reference databases. The reference database for taxonomic classification was tailored to *bacterial* protein and nucleotide genome sequences and indexed using Kaiju and Kraken2 respectively. Four indexed classifier-database combinations were created: kaiju-MAR (25 GB), kraken2-MAR (49 GB), Kaiju-RefSeq (31 GB), and kraken2-RefSeq (42.6 GB). Classified reads were imported using the phyloseq package [18] in R, and the corresponding count values were converted into percentages for both sequencing platforms (12 samples), where 100% refers to the total read count of a sample. Subsequently, the taxonomically classified reads were subjected to Principal Coordinate Analysis (PCoA) analysis using Bray-Curtis dissimilarities at the taxonomic ranks phylum, order, and genus in R with the ordinate function from phyloseq package.

### Assembly

The assembly of both normalized and preprocessed/clean reads from both HiSeq3000 and BGISEQ-500 platforms was performed using MEGAHIT v1.2.9 [19] and metaSPAdes v3.13.0 [20], except for the assembly of normalized reads from HiSeq3000 due to high computational requirements. It resulted in 84 combinations of input reads, assemblers, sequencing platforms, and sample points, and each combination was assembled at default k-mer values. Assembly qualities of contigs greater than 500 bp were evaluated using MetaQuast v5.0.2 [21]. The MEGAHIT assemblies were conducted locally using 40 threads on Intel(R) Xeon(R) Gold 6150 CPU @ 2.70 GHz processors and Intel(R) Xeon(R) Gold 6240R CPU @ 2.40 GHz processors. The metaSPAdes v3.13.0 assemblies were performed on Sigma2 - the National Infrastructure for High Performance Computing and Data Storage in Norway. Supplementary Fig. 1 was generated on relative values (–1 to 1) using the *ComplexHeatmap* R package [22]. Relative values were recomputed as mean of assembly statistics from each UnPreprocessed reads-assembler combination relative to average value obtained from all combinations, as described in a previous publication [23]. An in-house bash script was used to calculate the maximum memory (RAM) utilized and total run-time during both taxonomy classification and assembly.

## Results

### Normalization and preprocessing of sequence data

Illumina HiSeq3000 generated a higher number of bases, on average (~36 billion bases and fewer reads ~241 M) than BGISEQ-500 (~32 billion bases or ~322 M reads) at most sample points (Fig. 2). Normalization enabled us to retain ~ 23-38 billion high-quality bases with average Q20 scores of ~99.04% and ~97.25% for HiSeq3000 and BGISEQ-500 generated normalized/unprocessed reads, respectively (Supplementary Table 1). High-quality sequences were characterized by a lack of ambiguous base calls or base-calling errors, represented by an ‘N’. We observed that such errors were almost absent (<0.6%) in BGISEQ-500 reads but ranged from 5-11% at the end of HiSeq3000 reads (8 bp), which were discarded by quality control tools. Moreover, HiSeq3000 reads showed a high amount of Nextera adapter contamination in three out of 12 samples, while the BGISEQ-500 reads were almost free from adapter sequences. The duplication ratio in HiSeq3000 generated reads was slightly higher than that in BGISEQ-500 generated reads (Supplementary Table 1). The GC percentage of HiSeq3000 and BGISEQ-500 generated reads ranged between 52%–56% and 51%–55%, respectively (Supplementary Fig. 2). Finally, the length of preprocessed reads varied between 117–130 bp for HiSeq3000 and ~100 bp for BGISEQ-500.

### Taxonomic classification

The taxonomic classification of preprocessed reads from both sequencing platforms in all samples resulted in 16,667 unique *bacterial* taxonomic IDs (Supplementary Table 2.1). A significant proportion of taxa (~40.3%) were unique to a particular database, irrespective of the classifier and database type used (Supplementary Fig. 3). The RefSeq database uniquely identified approximately 49.5% (or 8,242 taxa) of total classified taxa. Similarly, the MAR database uniquely contributed significantly to the identification of approximately 40.7% (or 6,796 taxa) of the total taxa. Kraken2, using RefSeq, identified 1,066 taxa (6.4% of total classified taxa) that were unique and not identified by any other classifier-database combination. In contrast, 1,484 taxa or 8.9% of the total classified taxa were identified by all classifier-database combinations.

**Fig. 3.**
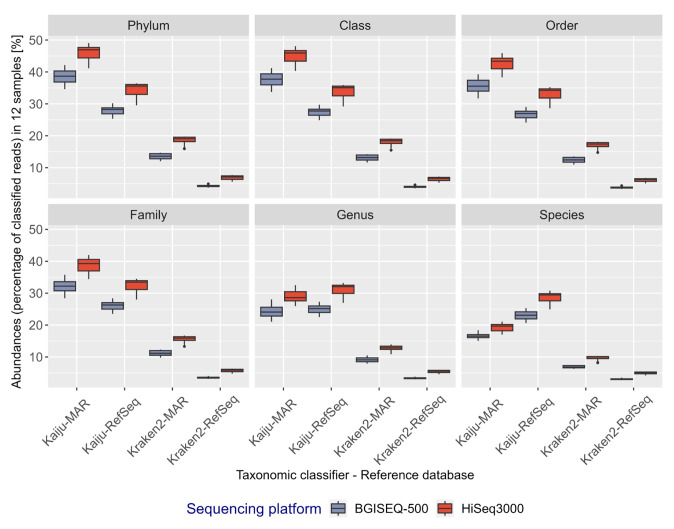
Percentage distribution of classified *bacterial* reads in 12 metagenomics samples at respective taxonomic ranks shown as box plots. Each box plot represents a combination of classifiers, sequencing platforms, and databases, represented in red (HiSeq3000) or blue (BGISEQ-500). Classified reads are displayed as percentages and grouped using R (*phyloseq*).

A decline in the percentage of taxonomically classified reads was observed at lower taxonomic ranks, regardless of the sequencing platform, classifier, or database used (Fig. 3). Across all taxonomic ranks and for each sample point, a higher fraction of HiSeq3000 reads were classified relative to BGISEQ-500 reads (average difference of ~1.93%–7.23%), irrespective of reference databases used (Supplementary Table 2.2). Kaiju classified a higher fraction of reads than Kraken2 for all taxonomic ranks (average difference of 22%–26.8% from *domain to family*, and 9.62%–23.58 % from *genus* to *species*) (Supplementary Table 2.3). The use of the curated marine-specific database (MAR) resulted in more reads being taxonomically classified than RefSeq (average difference of ~6.2%–11.7% from *domain to family*), except with Kaiju at *genus* and *species* level (Supplementary Table 2.3). However, at the taxonomic rank of *genus* and *species*, the percentage of reads classified using Kaiju-MAR declined more than that using Kaiju-RefSeq (average difference of ~1.4%–7.9%) (Fig. 3, Supplementary Table 2.3).

**Fig. 4.**
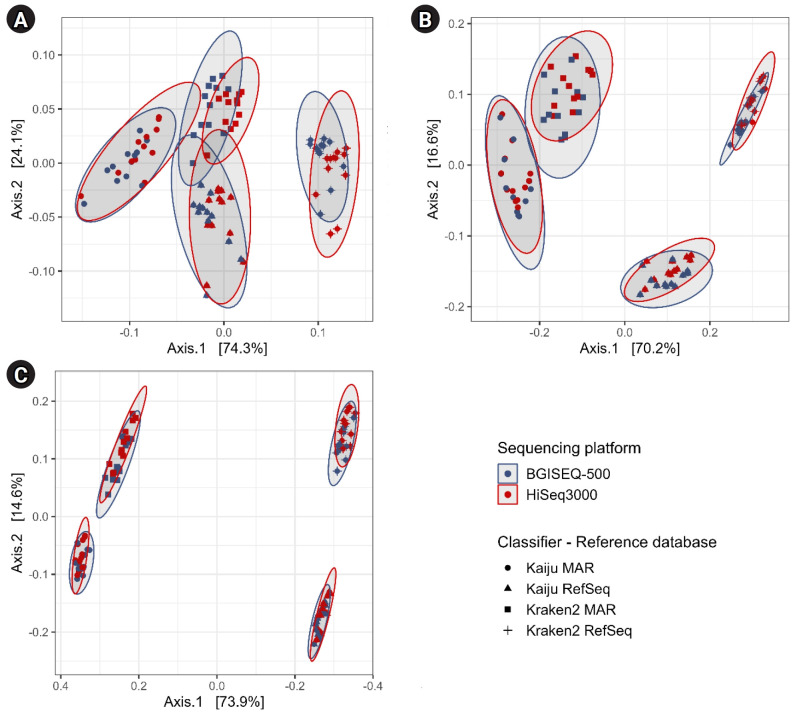
Principal coordinate analysis plot of taxonomically classified reads using the Bray-Curtis distance metric at taxonomic ranks: (A) phylum, (B) order, and (C) genus (x-coordinate flipped). Gray oval cluster each classifier-reference database combination at 95% confidence interval.

The PCoA of taxonomic profiles showed a clear clustering that correlates to the choice of reference databases and taxonomic classifiers. Approximately 73.9% of variation in the dataset is explained by the choice of reference database, while 14.6% can be attributed to the choice of taxonomic classifier at the *species* level (Fig. 4). At higher taxonomic ranks, there were no clear separations between the selected reference databases and taxonomic classifiers. The sequencing technology does not seem to contribute to the variations in ordination plot.

### Assembly

**Fig. 5.**
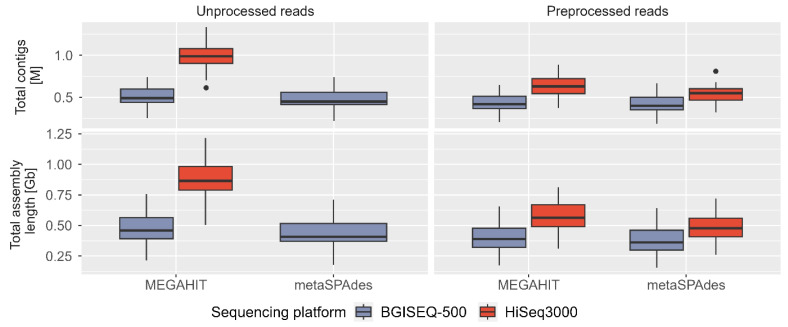
Boxplot showing assembly statistics using metaSPAdes and MEGAHIT on unprocessed and preprocessed reads from HiSeq3000 (red) and BGISEQ-500 (blue) sequencing platforms.

We assembled unprocessed and preprocessed/clean reads using two assemblers (MEGAHIT and metaSPAdes) independently, resulting in a total of 84 assemblies from seven categories (un/preprocessed reads, sequencing technology, and assemblers) (Supplementary Table 3, Supplementary Fig. 1). On average, MEGAHIT with HiSeq3000 unprocessed reads generated the largest and most contiguous assemblies (~866 Mb or ~976 kb contigs), while metaSPAdes using clean BGISEQ-500 reads produced the smallest assemblies (~371 Mb or ~415 kb contigs) (Fig. 5). Both assemblers consistently produced larger assemblies using HiSeq3000 reads, with an average difference of ~390 Mb (MEGAHIT using unprocessed reads), ~164 MB (MEGAHIT using clean reads), and ~116 MB (metaSPAdes using clean reads) compared to BGISEQ-500 reads. The largest contigs in all assembly categories ranged between 35–155 kb, except for outliers at ~192 kb (Supplementary Fig. 4A). The GC percentage of assemblies was ~53%–57% for HiSeq3000 and ~48%–56% for BGISEQ-500 (Supplementary Fig. 4B). The N50 contig length (>500 bp) remained similar (~0.75–1 kb) in all assemblies, irrespective of assemblers (Supplementary Fig. 4C).

### Computational requirements

The computational resources required for classification and assembly were minimally affected by the sequencing platform. The taxonomic classifier Kaiju efficiently utilized all available resources, while Kraken2 used approximately 35.58% of the CPU but classified the dataset 12 times faster than Kaiju (Supplementary Fig. 5A and 5B). Peak memory usage for Kaiju varied between ~28–35 GB and ~42–43 GB for Kraken2. MetaSPAdes required significantly higher memory (~515–554 GB) than MEGAHIT (~54–63 GB) for assembling the largest dataset (~32–38 billion bases) while for the smallest dataset (~19–23 billion bases), metaSPAdes and MEGAHIT used ~300 GB and ~35 GB of maximum memory (Supplementary Fig. 5D). Peak memory usage and run-time for all assemblies averaged approximately 428 GB or 37.22 h for metaSPAdes, and 46 GB or 6.48 h for MEGAHIT. The assembler run-time performance improved significantly using preprocessed or clean reads by ~45% (MEGAHIT using HiSeq3000), ~25% (MEGAHIT using BGISEQ-500), and ~31% (metaSPAdes using BGISEQ-500) compared to unprocessed reads (Supplementary Table 3).

## Discussion

Our study compared the metagenomic DNA sequences generated by two sequencing platforms, HiSeq3000 and BGISEQ-500, from 12 samples collected along the Norwegian coast. To evaluate the quality of the generated datasets, we utilized two different reference databases, taxonomic classifiers and assemblers.

Both platforms generated an unequal number of reads and base counts, which can vary due to factors such as microbial abundance, sequencing depth, and GC biases [24]. Library preparation methods specific to each platform can impact read duplication and adapter contamination [25]. There is also evidence of a platform-dependent GC distribution pattern between the BGISEQ-500 and HiSeq4000 [7]. To mitigate these limitations and differences, an equal number of bases were extracted from each sample point from both platforms, to normalize the data and facilitate further comparisons. Our analysis encompasses the evaluation of the percentage of reads classified at various taxonomic ranks, assembly statistics, and computational requirements for each platform's generated sequencing reads.

Our findings indicate that despite the differences in sequence length (HiSeq3000: 150 bp PE and BGISEQ-500: 100 bp PE), the base quality between these two sequencing platforms is comparable. The absence of adapter sequences in the BGISEQ-500 sequences resulted in a smaller proportion of reads being discarded during subsequent processing stages (Supplementary Table 1). However, a higher percentage of taxonomically classified reads was obtained from the HiSeq3000 platform, with a difference ranging from 1.93% to 7.23% on average, compared to the BGISEQ-500 platform, when evaluated across different taxonomic classifiers and reference databases (Fig. 3, Supplementary Table 2.1). This discrepancy could be due to a combination of factors, including sequence length, classification algorithms, and reference databases used. Previous research using short Illumina reads indicates a weak correlation between read length and classification success (the probability of correct classification out of total taxonomically classified reads) and for *bacteria* it remained relatively constant across different read lengths (100 bp and 150 bp) [26]. Also, Illumina short reads (100 bp and 150 bp) were found to have a constantly higher overall recall (the probability of correct classification out of total reads) for *bacteria* using Kraken2 [26]. The difference in the fraction of reads classified between the HiSeq3000 and BGISEQ-500 platforms could be attributed to differences in the classification method employed. Both Kaiju and Kraken2 utilize different approaches to taxonomically classify reads; Kaiju uses a maximum number of exact matches, while Kraken2 identifies fixed-size k-mers of variable length in reads and matches them against indexed databases. Although Kaiju had a slightly longer run-time, it efficiently utilized all computational resources and classified a higher percentage of reads (as shown in Supplementary Fig. 4A). Unfortunately, it was impossible to calculate the accuracy of the taxonomic classifiers used in this study as it requires simulation studies on known mock communities using reference databases, which was beyond the scope of this paper [11].

Our analysis showed that using both MAR and RefSeq protein databases was more effective in classifying reads compared to their nucleotide counterparts (Supplementary Table 2.3). This disparity could be attributed to the fact that protein sequences are more robust to nucleotide substitutions and sequencing errors [27]. Conversely, the nucleotide database showed a difference of 11–24 Gb in bacterial genome records compared to the protein counterpart. Moreover, using the RefSeq database (*bacteria*) as a reference led to a higher maximum memory requirement, possibly due to database composition and diversity (Supplementary Fig. 5C). Furthermore, the PCoA plot presented in Fig. 4 provided additional evidence of substantial differences at lower taxonomic ranks between the reference databases. The distinct clustering patterns strongly correlates with the selection of reference databases. Taken together, these results suggest that the taxonomic classification of sequence reads is primarily influenced by the choice of reference database, followed by the taxonomic classifiers and sequencing platforms used. Notably, the PCoA plot illustrates that the two sequencing technologies produced comparable taxonomic profiles.

As expected, the fraction of classified reads declines from the highest (*domain*) to the lowest (*species*) taxonomic rank (Fig. 3). The decline is due to a combination of the incomplete annotation of entries in the databases at lower taxonomic ranks (e.g., an entry can have higher taxonomic information e.g., only at *domain to family* rank), and that the classification algorithms fail to classify reads at lower ranks if reads matches equally well to multiple entries. Similar misclassification at the *genus* or *species* level have been previously reported due to bioinformatics contamination, instances where species having a higher average nucleotide identity than the true species [25] or the absence of closely related genomes in RefSeq which is rare at higher taxonomic ranks [28]. However, Kaiju address this by utilizing LCA method in cases of equally good matches to multiple taxa, leading to assignment of reads at a higher taxonomic rank.

As an example, taxonomic classification using Kaiju against the MAR database gradually declines from domain level with 100% classification to 71% at the family level. However, it drops significantly to 59.27% at the genus level and further to 43.09% at the species level. This drop in classification accuracy could be due to the presence of marine MAGs, SAGs, and incomplete genomes in the MarDb database [14], which is a component of MAR database. MAG and SAG genomic sequences are assembled from environmental samples and often contain fragments from multiple genomes or exhibit gaps and errors due to the complexities of assembling genetic material from diverse source. The high degree of fragmentation in MAGs and the presence of unknown microbes pose challenges in accurately classifying these microbes, especially at lower taxonomic ranks. The absence of representative taxon nodes for MAGs in the database can further complicate the process.

Although, Kaiju using RefSeq protein database, identified approximately 49.5% of the total identified taxa or 8,242 taxa, it remains uncertain whether these classified organisms are exclusively of marine origin. The difference in classified reads using Kaiju-MAR or Kaiju-RefSeq at lower taxonomic rank could result from non-marine strains in RefSeq database, fewer marine bacteria being taxonomically identified at species level in the MAR database or allocation of equally classified reads to higher taxonomic rank using the LCA algorithm by Kaiju. Nevertheless, despite these challenges, Kaiju consistently demonstrated the highest recall with HiSeq3000 generated sequences, except at *species* level, where the MAR database proved most effective for marine metagenomics samples.

Assessing the quality of metagenomics assemblies can be challenging due to the absence of reference genomes representing diverse communities. Our comparative analysis using same base count demonstrated that both assemblers produced larger assembly lengths and total contigs using preprocessed HiSeq3000 reads in majority of sample points. The BGISEQ-500 assemblies exhibited slightly better N50 and L50 contig values, although these statistics can be easily manipulated due to being based on the ordered length of contigs. In addition to our assessment of assembly quality, we compared assemblers which revealed that MEGAHIT generated larger assembly lengths and longer total contigs than metaSPAdes, with an average difference of 2.56%–18.74% and up to 16.55% (using unprocessed BGISEQ-500 reads), 8.5%–19.15% and 6.82%–19.29% (using clean HiSeq3000 reads), and up to 13.49% and upto 11.65% (using clean BGISEQ-500 reads) in all 12 samples (Fig. 5). We employed de Bruijn graph (dBg) based assemblers, which require a selection of k-mer size and can significantly impact the final assembly (Fig. 5, Supplementary Fig. 4C and 4D). The choice of k-mer size influences the complexity of the graphs and affects the ability to resolve repeats, errors, and heterozygosity in the assembly [29]. Choosing a smaller k-mer size results in more connected graphs, while a larger k-mer size leads to simplified graphs. In our study, we used the recommended default k-mer size to obtain assembly statistics across different sequencing technologies, processed reads, and assembly programs. While both MEGAHIT and metaSPAdes performed comparably in terms of assembly, MEGAHIT was more resource-efficient overall. The peak memory usage for MEGAHIT to assemble 200 million reads or approximately 31 billion bases was 63 GB, making it a preferred option for memory-intensive metagenomics projects.

Our findings suggest that both platforms are capable of generating high-quality metagenomic data, but there were some notable differences in their performance. Overall, the choice of sequencing platform should depend on the specific research question and the characteristics of the microbial community being studied. Our study provides valuable insights into the performance of two popular platforms, which can aid researchers in making prior decisions about their sequencing strategies.

The study compared the results of two short read sequencing platforms, HiSeq3000 and BGISEQ-500, and we found that they produced comparable results. We also compared different sequencing technologies, taxonomic classifiers, reference databases and assemblers. The findings show that short read sequencing platforms can be used interchangeably in metagenomics studies, without compromising result quality. Our studies show that each sequencing platform has strengths and weaknesses; therefore, the selection of specific platform should be based on the specific research questions and experimental design. For metagenomics analysis the choice of reference database is more essential for taxonomic classification where the sequencing method itself becomes less significant. Finally, taxonomic classifiers and assembly tools have different computational requirements and the availability of resources needs to be taken into account.

## Supplementary Materials

Supplementary Table. 1. Sequencing reads statistics at different stages of processing steps. Reads were normalized or downsampled on the basis of equal base count. The reference base count for each sample point base was chosen based on minimum base count among genereated sequences per sample point

Supplementary Table 2.1. Taxonomy table showing the number of reads classified for a specific sample using a particular combination of classifier and reference database

Supplementary Table 2.2. Percentage of reads classified in a sample at different taxonomic levels using a particular combination of classifier and reference database

Supplementary Table 2.3. Comparative table showing effect of reference database MAR and RefSeq on average percentage of classified reads using same classifier Kaiju and Kraken2

Supplementary Table 3. Assembly statistics. Contigs >500 bp are shown here. For N50 and L50 calculation, both contigs length >500 and > 1000 bp are calculated

Supplementary Fig. 1. Heatmap showing assembly statistics (>500 bp contigs length) and computational memory required by two assemblers using before and after preprocessed sequence read generated from two sequencing platforms (ComplexHeatmap in R). Relative values were computed as mean of assembly statistics from each Unpro\Preprocessed reads-assembler combination relative to average value obtained from all combinations and further color coded based on comparative performance from red block (best) to white block (better) to blue block (good).

Supplementary Fig. 2. GC content (%) computed at different stages of reads preprocessing steps.

Supplementary Fig. 3. Venn diagram showing 16,667 bacterial taxa identified by using four combinations of classifier and reference database on twelve metagenomic preprocessed sequence reads generated from HiSeq3000 and BGISEQ-500 sequencing platforms. Uniquely identified bacterial taxon from each of the four combinations was used to generate an overlapping Venn diagram using Venny 2.1.0.

Supplementary Fig. 4. Boxplot displaying assembly statistics by two assemblers on twelve metagenomes using HiSeq3000 and BGISEQ-500 unprocessed and preprocessed sequence reads. BGISEQ-500 generated reads sequences are represented in blue, while HiSeq3000 in red. Statistical transformation of data was performed using the default geom_boxplot option in R (ggplot2). (A) Largest contig. (B) GC (%) of assemblies. (C) N50. (D) L50.

Supplementary Fig. 5. Boxplot depicting computational resource usage in terms of memory and time required by two taxonomic classifiers (A–C) and assembly (D). All results are from twelve metagenomic reads sequences generated from two sequencing platforms. BGISEQ-500 generated reads sequences are shown in blue, while HiSeq3000 is in red. Statistical transformation of data was performed using the default geom_boxplot option in R (ggplot2). (A) CPU resources utilization (Taxonomic classification). (B) Total Run-Time (Taxonomic classification). (C) Peak Memory usage (Taxonomic classification). (D) Peak Memory usage (Assembly).
