# Supplementary material for: Comparative analysis of HiSeq3000 and BGISEQ-500 sequencing platform with shotgun metagenomic sequencing data
Source: Genomics Inform. 2023 Dec 29;21(4):e49. doi: 10.5808/gi.23072 (PMC10788357; doi:10.5808/gi.23072)
Supplement: Supplementary Fig. 1. — Heatmap showing assembly statistics (>500 bp contigs length) and computational memory required by two assemblers using before and after preprocessed sequence read generated from two sequencing platforms (ComplexHeatmap in R). Relative values were computed as mean of assembly statistics from each Unpro\Preprocessed reads-assembler combination relative to average value obtained from all combinations and further color coded based on comparative performance from red block (best) to white block (better) to blue block (good). [file gi-23072-Supplementary-Fig-1.pdf]

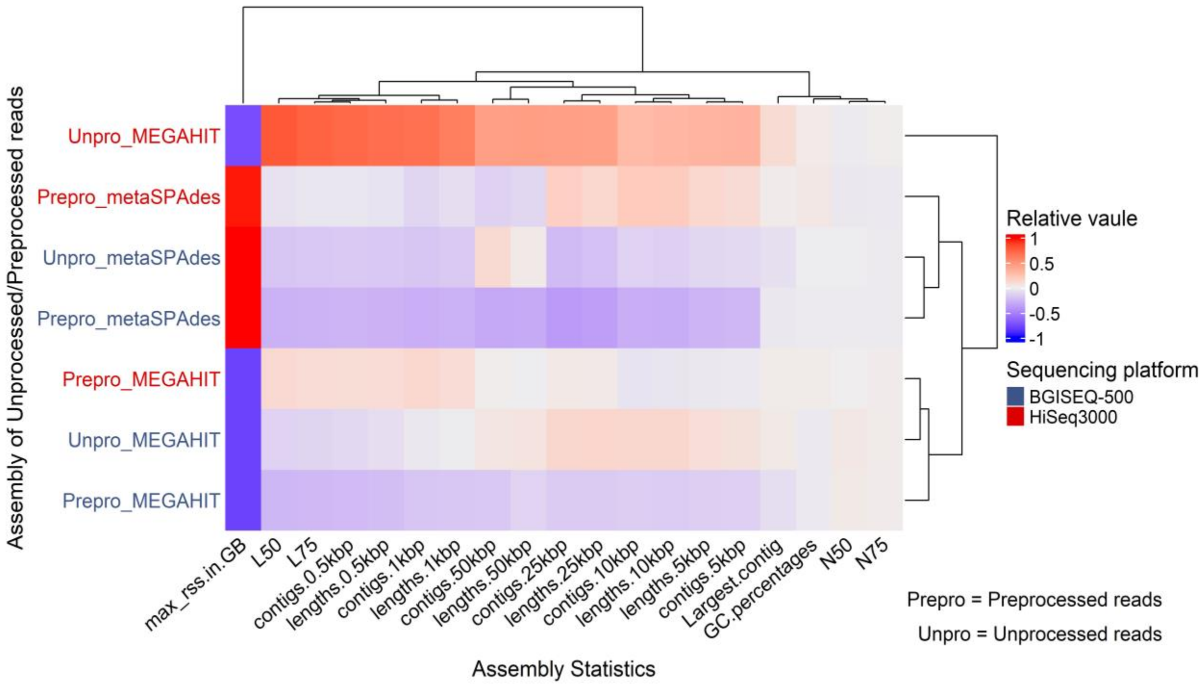

Supplementary Figure S1: **Heatmap showing assembly statistics (> 500 bp contigs length) and computational memory required by two assemblers using before and after preprocessed sequence read generated from two sequencing platforms** (*ComplexHeatmap* in R). Relative values were computed as mean of assembly statistics from each Unpro\Preprocessed reads-assembler combination relative to average value obtained from all combinations and further color coded based on comparative performance from red block (best) to white block (better) to blue block (good).
