# Supplementary figures and images for: Comparative analysis of HiSeq3000 and BGISEQ-500 sequencing platform with shotgun metagenomic sequencing data
Source: Genomics Inform. 2023 Dec 29;21(4):e49. doi: 10.5808/gi.23072 (PMC10788357; doi:10.5808/gi.23072)

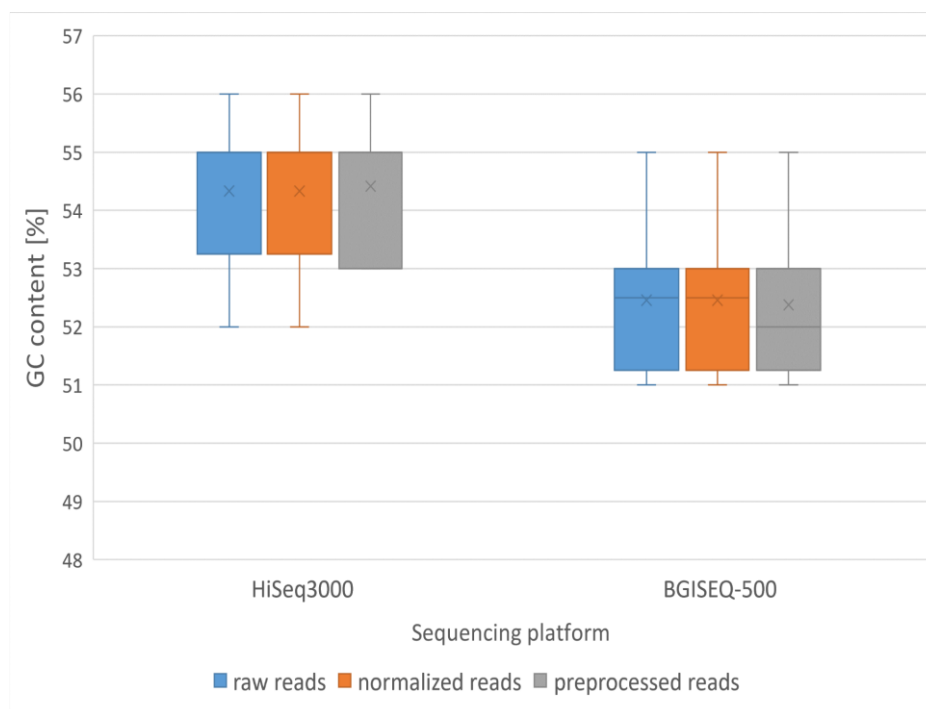

**Supplementary Figure S2:** GC content (%) computed at different stages of reads preprocessing steps.

Supplement: Supplementary Fig. 2. — GC content (%) computed at different stages of reads preprocessing steps. [file gi-23072-Supplementary-Fig-2.pdf]
