# Supplementary material for: Comparative analysis of HiSeq3000 and BGISEQ-500 sequencing platform with shotgun metagenomic sequencing data
Source: Genomics Inform. 2023 Dec 29;21(4):e49. doi: 10.5808/gi.23072 (PMC10788357; doi:10.5808/gi.23072)
Supplement: Supplementary Fig. 3. — Venn diagram showing 16,667 bacterial taxa identified by using four combinations of classifier and reference database on twelve metagenomic preprocessed sequence reads generated from HiSeq3000 and BGISEQ-500 sequencing platforms. Uniquely identified bacterial taxon from each of the four combinations was used to generate an overlapping Venn diagram using Venny 2.1.0. [file gi-23072-Supplementary-Fig-3.pdf]

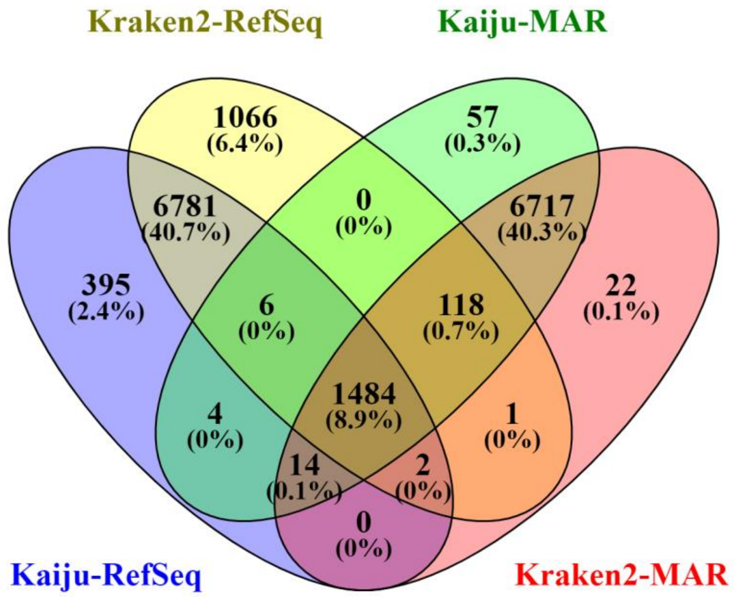

Supplementary Figure S3: **Venn diagram showing 16,667 bacterial taxa identified by using four combinations of classifier and reference database on twelve metagenomic preprocessed sequence reads generated from HiSeq3000 and BGISEQ-500 sequencing platforms**. Uniquely identified bacterial taxon from each of the four combinations was used to generate an overlapping Venn diagram using *Venny 2.1.0*
