# Supplementary material for: Comparative analysis of HiSeq3000 and BGISEQ-500 sequencing platform with shotgun metagenomic sequencing data
Source: Genomics Inform. 2023 Dec 29;21(4):e49. doi: 10.5808/gi.23072 (PMC10788357; doi:10.5808/gi.23072)
Supplement: Supplementary Fig. 4. — Boxplot displaying assembly statistics by two assemblers on twelve metagenomes using HiSeq3000 and BGISEQ-500 unprocessed and preprocessed sequence reads. BGISEQ-500 generated reads sequences are represented in blue, while HiSeq3000 in red. Statistical transformation of data was performed using the default geom_boxplot option in R (ggplot2). (A) Largest contig. (B) GC (%) of assemblies. (C) N50. (D) L50. [file gi-23072-Supplementary-Fig-4.pdf]

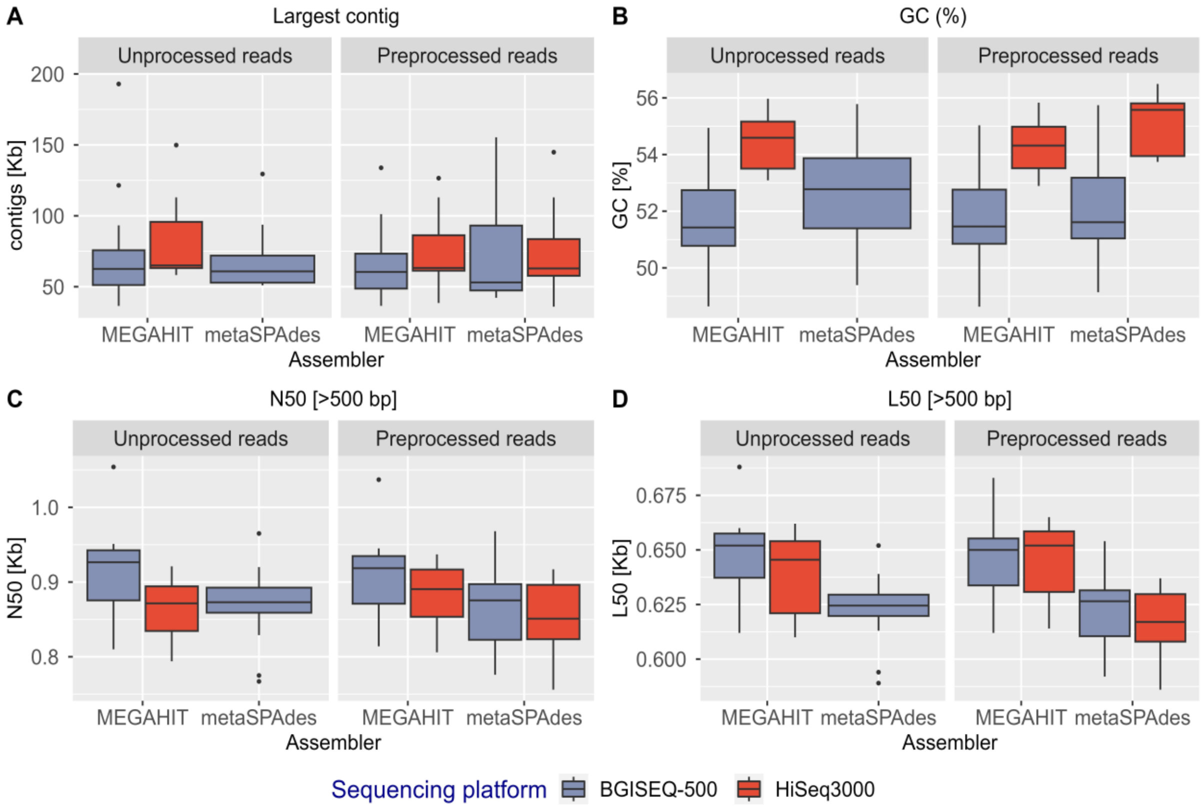

Supplementary Figure S4: **Boxplot displaying assembly statistics by two assemblers on twelve metagenomes using HiSeq3000 and BGISEQ-500 unprocessed and preprocessed sequence reads**. BGISEQ-500 generated reads sequences are represented in blue, while HiSeq3000 in red. Statistical transformation of data was performed using the default *geom_boxplot* option in R (ggplot2). **A.** Largest contig, **B.** GC (%) of assemblies, **C.** N50. **D.** L50.
