# Supplementary material for: Comparative analysis of HiSeq3000 and BGISEQ-500 sequencing platform with shotgun metagenomic sequencing data
Source: Genomics Inform. 2023 Dec 29;21(4):e49. doi: 10.5808/gi.23072 (PMC10788357; doi:10.5808/gi.23072)
Supplement: Supplementary Fig. 5. — Boxplot depicting computational resource usage in terms of memory and time required by two taxonomic classifiers (A–C) and assembly (D). All results are from twelve metagenomic reads sequences generated from two sequencing platforms. BGISEQ-500 generated reads sequences are shown in blue, while HiSeq3000 is in red. Statistical transformation of data was performed using the default geom_boxplot option in R (ggplot2). (A) CPU resources utilization (Taxonomic classification). (B) Total Run-Time (Taxonomic classification). (C) Peak Memory usage (Taxonomic classification). (D) Peak Memory usage (Assembly). [file gi-23072-Supplementary-Fig-5.pdf]

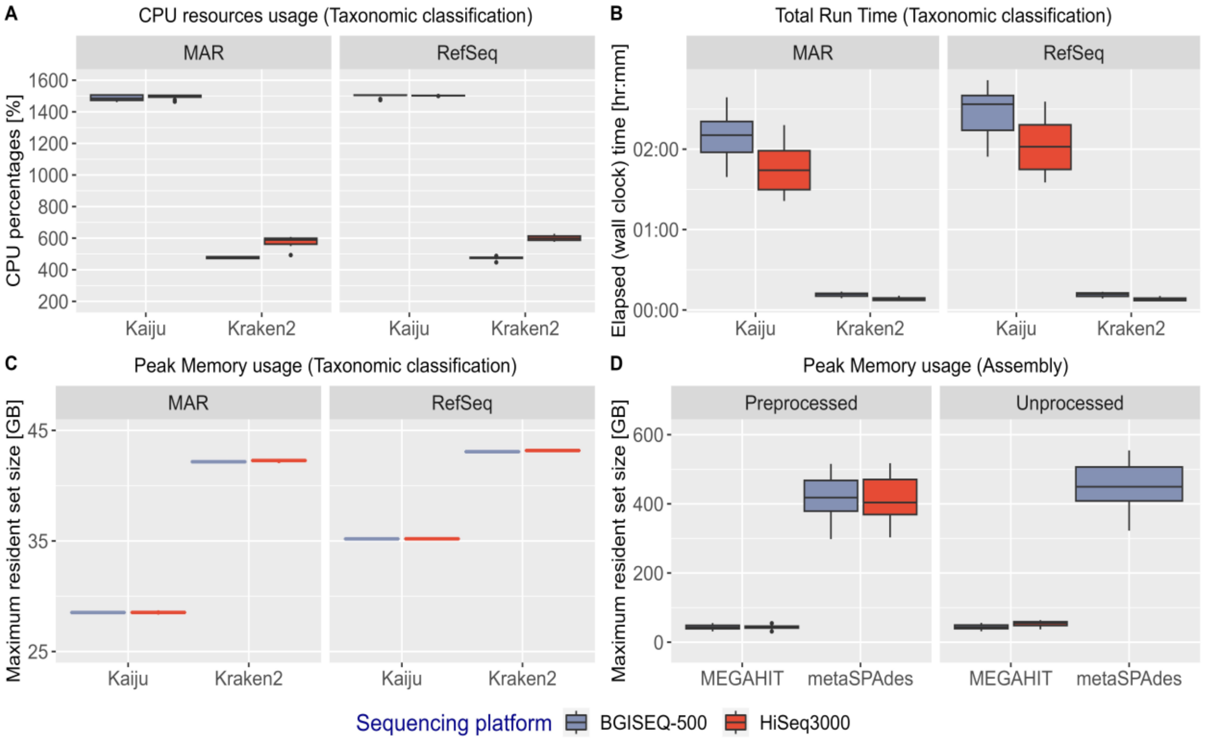

Supplementary Figure S5: **Boxplot depicting computational resource usage in terms of memory and time required by two taxonomic classifiers (A-C) and assembly (D).** All results are from twelve metagenomic reads sequences generated from two sequencing platforms. BGISEQ-500 generated reads sequences are shown in blue, while HiSeq3000 is in red. Statistical transformation of data was performed using the default *geom_boxplot* option in R (ggplot2). **A.** CPU resources utilization (Taxonomic classification), **B.** Total Run-Time (Taxonomic classification), **C.** Peak Memory usage (Taxonomic classification), **D.** Peak Memory usage (Assembly).
